# Development of Air Quality Monitoring Systems: Balancing Infrastructure Investment and User Satisfaction Policies

**DOI:** 10.3390/s25030875

**Published:** 2025-01-31

**Authors:** Olga Sokolova, Anastasia Yurgenson, Vladimir Shakhov

**Affiliations:** 1 The Artificial Intelligence Research Center of Novosibirsk State University, 630090 Novosibirsk, Russia; nastya@rav.sscc.ru; 2 Institute of Computational Mathematics and Mathematical Geophysics, 630090 Novosibirsk, Russia; shakhov@rav.sscc.ru

**Keywords:** air quality, environmental monitoring, air pollution detection, artificial intelligence, cluster number optimization, Lambert W function, air pollution monitoring, sensors, smart city

## Abstract

Air quality monitoring is a critical aspect of urban management. While poor air quality negatively impacts public health and well-being, implementing effective monitoring systems often involves significant costs. This paper addresses the optimization of air quality monitoring systems by balancing cost-effectiveness with citizen satisfaction. The core objective is to identify an optimal trade-off between user satisfaction and budgetary constraints. To achieve this, we optimize the number of clusters, where each cluster represents a group of users served by the nearest air quality sensor. Additionally, we present a penalty function that emphasizes prompt air pollution warnings, facilitating preventive actions to reduce exposure to polluted areas while ensuring a cost-effective solution. This approach enables the formulation of well-founded performance requirements for AI-driven algorithms tasked with analyzing air quality data. The findings contribute to the development of efficient, user-centric air quality monitoring systems, highlighting the delicate balance between infrastructure investment, AI algorithm efficiency, and user satisfaction.

## 1. Introduction

Air pollution refers to changes in the atmosphere’s properties due to the introduction of various harmful substances and compounds, which negatively impact the environment. The primary sources of air pollution include industrial activities, motor vehicle emissions, and forest fires. The presence of pollutants in the air poses significant health risks and contributes to a range of diseases, particularly in large urban areas. The World Health Organization has repeatedly identified air pollution as one of the greatest threats to human health, resulting in millions of deaths each year [1,2]. Consequently, monitoring air quality is crucial for environmental management and for establishing scientifically grounded air quality standards. To obtain accurate information on air quality with high spatial and temporal resolution, extensive monitoring of atmospheric pollutants is essential [3].

Research in this area can lead to the development of effective strategies for reducing harmful emissions and enhancing public health policies. For instance, understanding the health impacts of particulate matter could prompt the establishment of stricter regulations for industries that are significant sources of air pollution. However, the technologies currently employed for air quality monitoring often do not meet the growing demands. To implement effective policies aimed at improving air quality in megacities, modern monitoring systems must be utilized. This will enable authorities to quickly assess compliance with environmental quality standards and take corrective actions when violations occur. For example, if monitoring data indicates elevated pollution levels in a specific area, project developers can prioritize public transportation initiatives to reduce vehicle emissions.

Detecting air pollution in large cities necessitates a substantial number of air quality monitoring stations. However, the deployment and maintenance of such stationary stations can be prohibitively expensive. In recent years, a combination of stationary and mobile monitoring stations has been adopted in megacities, utilizing both fixed sensors and those mounted on vehicles [4]. Wireless sensor networks facilitate real-time air quality analysis, allowing for prompt responses to any changes. Recent publications have documented the implementation of such monitoring systems across various countries, including the UK, Russia, South Korea, China, and others. A comparative analysis of modern monitoring systems is presented in the article [5].

To evaluate the effectiveness of contemporary monitoring systems, it is important to consider factors such as sensor placement density, sensor characteristics, and the communication protocols used for data transmission. Optimizing sensor placement is particularly crucial for identifying pollution sources and providing accurate air quality data to inform the public. This involves positioning sensors near pollution sources and in areas with vulnerable populations, such as schools, hospitals, and nursing homes, to ensure timely dissemination of information to citizens. One paper [6] addressed the challenges of sensor placement, aiming to: (1) enhance the assessment of vulnerable individuals’ exposure to air pollution; (2) maximize public satisfaction with the information provided about air quality; and (3) improve monitoring of emissions from transportation. The authors proposed effective solutions for each of these challenges.

The accuracy of information from wireless sensors is generally lower than that of devices used in monitoring stations [7,8,9]. To enhance the efficiency of sensor systems, it is essential to improve sensor hardware and develop advanced data collection and transmission algorithms. The integration of the Internet of Things (IoT) and artificial intelligence (AI) into these algorithms significantly enhances the accuracy and speed of pollution source detection [10]. In [11], the authors discuss the implementation of virtual sensors that leverage AI and IoT capabilities. These virtual sensors estimate pollutant values for which physical sensors are unavailable, utilizing data from other pollutants and their correlations [12]. Additionally, AI algorithms can predict air quality parameters before they reach hazardous levels, enabling timely interventions.

This paper focuses on optimization strategies for air quality monitoring systems. We refine a citizen-centric approach validated in Cambridge to model user satisfaction, which diminishes as the distance from the user to the nearest sensor increases, employing an exponential decay function [6]. Users are clustered based on their proximity to sensors, reflecting their reliance on localized environmental data. Optimizing user satisfaction necessitates a balance between the number of sensors and the associated system costs; while more sensors can reduce the cluster radius, they also increase expenses. To tackle this, we formulate a nonlinear optimization problem using a profit function that incorporates both user satisfaction and system costs, deriving an analytical solution with the Lambert W-function.

Moreover, we address the challenges of pollutant detection stemming from the stochastic nature of pollution and the limitations of sensor hardware, assuming the application of AI-based algorithms to enhance detection accuracy. Given that collateral damage escalates with delays in pollution detection, optimizing monitoring systems often prioritizes the acceleration of data collection and processing [13,14]. Consequently, we analyze a penalty function optimization problem that reconciles pollution detection delays with operational costs. The solutions obtained illustrate the effectiveness of our enhanced approach in ensuring the efficiency and practicality of contemporary air quality monitoring systems.

The rest of this paper is organized as follows. Section 2 covers the related work, Section 3 describes the methodology, Section 4 presents the results, and Section 5 concludes the paper.

## 2. Related Works

The optimization of air pollution monitoring is a critical area of research, particularly as urbanization and industrial activities continue to impact air quality. Recent studies have identified industry as the primary contributor to air pollution in major metropolitan areas. For instance, a study examining air quality in Jakarta emphasized the urgent need for national policies aimed at environmental improvement [15]. Utilizing dynamic system modeling, the authors predicted that air pollution in Jakarta will deteriorate by the end of 2027, posing a significant threat to public health. They stressed the importance of reducing coal usage in power plants and other industrial sectors to enhance air quality.

The study in [16] presented a methodology for determining the optimal number of air quality monitoring stations within a network. This approach utilizes triangular fuzzy numbers (TFN) to identify monitoring locations, with the network configuration based on the concept of a sphere of influence, which is defined using thresholds for spatial correlation coefficients between pollutant concentrations at monitoring stations. Another study [17] addressed the optimal distribution of monitoring stations by considering transportation patterns and spatial factors such as population density and emission sources. It evaluated three quality indicators: accuracy gain, information gain, and degrees of freedom for the signal, all of which are derived from the singular values of the sensitivity matrix that characterizes the network’s accuracy.

Researchers have noted that contemporary air quality monitoring methods incorporate both technological advancements (including various types of monitoring stations and sensors) and artificial intelligence to facilitate data collection and transmission from numerous sensors [18,19,20,21,22,23,24,25,26].

The study in [18] explored the establishment of a comprehensive air quality monitoring network within a chemical industrial park in Shanghai, China. It introduced an observation performance score that integrates averages from pollution detection effectiveness and source identification accuracy. To improve computational efficiency, a novel ranking method was developed, combining Gaussian matching models with source area analysis.

The study in [19] presents a new approach to designing monitoring networks using an optimization method. First, random selection of sites for the placement of monitoring stations is performed, and then their spatial distribution in the study area is optimized. The authors used the spatial simulated annealing method.

The concentration of pollutants is influenced by numerous unpredictable factors that are challenging to account for. The paper [20] highlights the connection between traffic flow estimation and pollutant emission evaluation, emphasizing how vehicular traffic patterns impact air quality. This research addresses the selection of a subset of sensors from a larger pool for monitoring a transportation network. It introduces a simulated annealing heuristic to tackle the selection problem, with the chosen sensors subsequently used to construct a Luenberger observer, a mathematical tool in control theory designed to accurately estimate the internal state of a dynamic system based on its inputs and outputs.

In the paper [21], a route-based fragmentation model was employed to strategically distribute sensors across micro-regions with similar air pollution characteristics. This approach utilized a genetic algorithm to optimize sensor configuration, aiming to minimize data distribution variance. The use of spatial interpolation to estimate sensor outputs allowed for a more comprehensive understanding of pollution levels across the monitored area. Another paper [22] introduced an information theory-based method for optimal sensor placement, leveraging the Lagrange atmospheric dispersion model to assess air pollution levels under varying meteorological conditions. This study contrasted random sensor placement with a strategy focused on maximum cumulative pollution levels, emphasizing the importance of informed sensor deployment to enhance data quality.

Various methods are used to monitor air quality in large areas. Satellite methods (using satellite data to monitor the concentration of pollutants) and automatic monitoring methods (installing stationary and mobile monitoring devices) are most often used to assess the impact of pollutants on the environment. Combining these two methods allows the advantages of both satellite remote sensing and ground-based observations to be used. While ground-based monitors provide more precise measurements of air quality at a specific location in the immediate vicinity of the monitor, satellite sensors provide a much wider area of coverage [23].

The design problems of air quality monitoring networks are important in cases where different operating conditions occur. The paper [24] addressed the design of air quality monitoring networks under uncertain operational conditions, particularly focusing on the maximum weighted location coverage problem. The application of linear approximation methods to nonlinear models enhances computational efficiency, which is crucial for real-time monitoring and decision-making.

In the paper [27], the authors conducted a comprehensive review of literature on the design of air quality monitoring systems, highlighting that most research predominantly employs linear programming techniques, often overlooking the potential of multicriteria optimization. They emphasized the critical need to consider both the cost of the monitoring system and various efficiency indicators in tandem. Additionally, the authors pointed out the importance of addressing citizens’ information needs regarding air quality. As such, the design of modern air quality monitoring systems constitutes a multicriteria optimization problem. However, there remains a notable gap in the literature concerning methodologies that balance these competing criteria. To the best of our knowledge, no existing studies offer an analytical solution for optimizing the trade-off between system cost and user experience quality, nor do they address the optimization of key performance indicators, such as the time efficiency of data collection from monitoring systems, which is crucial for ensuring timely public notifications regarding air pollution.

Overall, these referenced studies underscore the importance of employing diverse methodologies and interdisciplinary approaches to effectively monitor and manage air quality in urban environments. Table 1 presents a brief systematic overview of these publications with a description of the methods and technologies used by the authors.

The relationship between urban traffic and air quality is explored in the studies in [25,26]. The article [25] analyzes the problem of road traffic in urban areas in Luxembourg and presents an analysis of air pollution data depending on road traffic. The authors studied the relationship, which evaluates the dependence of the effect of some pollutants on air temperature and humidity. In the paper [26], a statistical analysis was performed to investigate the influence of local road traffic and meteorological conditions on CO, N$O_{2}$, and C$O_{2}$ concentrations. Two different methods were applied: a linear regression model and an artificial neural network. As a result, the findings from paper [26] suggest that meteorological factors may play a more substantial role in influencing pollutant concentrations than local traffic, highlighting the complexity of air quality dynamics.

It is important to highlight several recent publications that examine the impact of air pollutants on human health [28,29,30,31]. The authors of these studies consistently emphasize that urbanization and the proximity of industrial facilities to residential areas contribute to poor local air quality, which in turn adversely affects public health. To assess health risks associated with air pollution, researchers utilize an assessment coefficient known as the Air Pollution Health Risk Assessment (AP-HRA). The authors of the article [28] note that the assessment of the AP-HRA index helps in making decisions in the field of public policy to improve the quality of health care. The authors describe how health hazards from emissions into the atmosphere are measured, as well as how the impacts associated with air pollution are quantified. Article [30] explores the impact of pollutants on public health through diverse mathematical approaches, considering multiple factors such as age, gender, and duration of exposure. The findings reveal that results can vary significantly depending on the methodology employed. Additionally, article [31] addresses the ongoing challenges related to assessing human health risks from air pollution. The authors highlight that air pollution remains a pressing issue in many countries, underscoring the need for enhanced environmental protection efforts. Therefore, air quality monitoring is a highly relevant topic, and any research in this area is vital for advancing public health initiatives.

Numerous studies have explored the application of machine learning methods to air pollution detection, demonstrating promising results. Among these, deep learning techniques, convolutional neural networks, and hybrid approaches have shown particularly high efficiency in identifying and analyzing pollution patterns. A systematic review of these techniques can be found in [32]. However, the pursuit of higher efficiency in air pollution detection often incurs significant overhead costs. These include increased computational requirements, higher storage demands, and additional expenses related to the transmission and processing of larger data volumes, among others. Despite the progress in enhancing detection capabilities, there is a notable gap in the development of approaches that facilitate balanced trade-off strategies between competing performance indicators. For instance, while faster detection of hazardous conditions is critical, it often comes at the expense of rising operational costs. The absence of well-defined frameworks to navigate these trade-offs underscores the need for future research that harmonizes the speed of detection with the associated overhead costs, enabling more sustainable and efficient air quality monitoring solutions.

## 3. Methodology

To formally assess the population’ s satisfaction with the air quality monitoring system, we employ the citizen-centric approach [6], which has been validated through testing in Cambridge. Observations indicate that individuals naturally refer to the nearest air monitoring station for environmental information. Consequently, a user’s satisfaction with an air monitoring system can be modeled as a function of their distance to the closest sensor. As the distance diminishes, users are more likely to align their activities with the monitoring results. Conversely, as the distance grows, the relevance of these results diminishes, eventually becoming negligible. Therefore, any function defined for all non-negative real numbers, G(d), taking non-negative values and monotonically decreasing to zero, can serve as an admissible satisfaction function. The variable *d* represents the distance between the user and the sensor. According to the mentioned approach, an exponential decay is used as the satisfaction function:

(1) $$G\left(d\right)=e^{-\vartheta d}$$

Here, the parameter ϑ serves as an exponential decay factor, determining the rate at which the satisfaction function decreases with distance. Lager values of ϑ produce a steeper decline. The default value of ϑ is set to one. Without loss of generality, we adopt this default assumption in our analysis.

**Figure 1:**
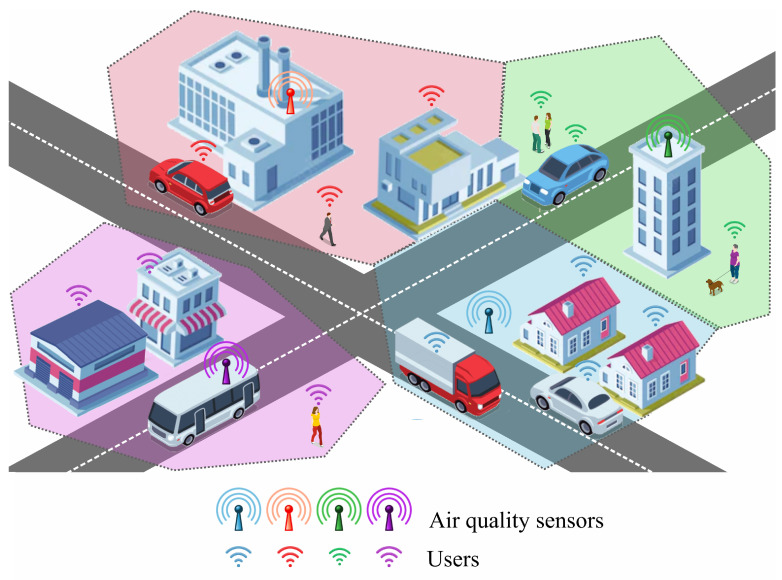
Clustering smart city residents based on their proximity to air quality sensors.

In this paradigm, users of the air quality monitoring system are grouped into clusters based on their proximity to the sensors, as shown in Figure 1. Each sensor defines a cluster that encompasses all users for whom that sensor is the closest. This clustering approach reflects the natural dependence of users on the nearest source of environmental data and divides users into disjoint groups that collectively cover the entire monitored area. However, the previous assumption that the distance *x* is the same for all users in the cluster seems impractical. Therefore, we consider this distance as a random variable characterized by the cumulative distribution function F(x) and the probability density function f(x). Since in general the distance is random, the satisfaction function (2) is also random. Therefore, we consider the mathematical expectation of the satisfaction function:

(2) $$G_{E}=\int_{-\infty }^{\infty }e^{-x}f\left(x\right)dx$$

Urban planning often aims to achieve a balanced distribution of residents, businesses, and public spaces around key infrastructure, including environmental monitoring systems. In smart city scenarios, it is therefore reasonable to assume a uniform distribution of users across the area monitored by a sensor. In recent publications, this assumption is typical for IoT scenarios [33,34,35]. A uniform distribution ensures that no specific area within the sensor’s range is disproportionately favored or overlooked, making it a practical and effective assumption for analysis when detailed user data is unavailable. Although scenarios such as population clusters near transportation hubs or commercial districts may arise, a uniform distribution provides a reliable approximation of real-world conditions in the absence of precise demographic or geographic information.

Thus, we assume that the cumulative distribution function of the distance between the customer and the nearest air quality sensor is as follows:

(3) $$F\left(x\right)=\left\{\begin{array}{c} 0,\;\;x<0\\ \frac{x}{R},\;0\leq x\leq R\\ 1,\;\;x>R \end{array}\right.\;$$

The parameter *R* denotes the cluster’s radius, encapsulating the maximum distance a customer may be from the nearest air quality sensor within the cluster. The mathematical expectation of the satisfaction function for a uniformly distributed distance according to (2) is as follows:

(4) $$G_{E}\left(R\right)=\frac{{1-e}^{-R}}{R}$$

The cost of an air monitoring system is usually linear with the number of sensors [36]. As the number of sensors increases, the cluster radius decreases, i.e., a sensor covers a smaller area. If the distances from the user to the sensors are independent and identically distributed random variables with a uniform distribution, then the expected minimum distance is directly proportional to the size of the monitoring area and inversely proportional to the number of sensors. Hence, we can assume that the cost of monitoring systems is calculated as follows:

(5) $$C_{S}\left(R\right)=\frac{S}{R}$$

Here, *S* is a given constant that is proportional to both the size of the monitoring region and the cost of the monitoring system.

The parameter *S* can directly reflect the size of the service area, and the structural cost expressions naturally follow from the property of the mathematical expectation of the minimum of i.i.d. uniformly distributed random variables. However, the formula remains applicable in more general scenarios. In such cases, an estimate for the parameter $\hat{S}$ can be obtained using regression analysis. Given a linear relationship between the dependent variable $\left(C_{S}\right)$ and the independent variable $1/R_{i}$, with data points derived from *K* experiments, a one-parameter linear regression is performed. Here, only the slope parameter is estimated (without the intercept). The estimation is conducted using the least squares method [37]:

$$\hat{S}=\frac{\sum_{i=1}^{K}\frac{C_{S}\left(R_{i}\right)}{R_{i}}}{\sum_{i=1}^{K}{\left(\frac{1}{R_{i}}\right)}^{2}}$$

where $C_{S}\left(R_{i}\right)$ are the observed values and $1/R_{i}$ are the corresponding observation points.

Our objective is to optimize user satisfaction while maintaining a reasonable cost, striving to maximize the following expression, which we shall refer to as the profit function:

(6) $$F_{P}\left(R\right)={w_{1}G}_{E}\left(R\right)-w_{2}C_{S}\left(R\right)$$

Here, the parameters $w_{1}$ and $w_{2}$ represent relative importance factors (weights) that balance the contributions of user satisfaction and system cost in the overall profit calculation. The weights $w_{1}$ and $w_{2}$ can be adjusted based on the air monitoring policy. A policy primarily focused on customer experience quality may assign a higher value to $w_{1}$. Conversely, the need to comply with budget constraints for the monitoring system may lead to an increase in the weight $w_{2}$. The selection of weight values in a specific scenario depends on the context, the objectives of the research, the availability of experimental data, and theoretical considerations. In situations requiring a trade-off between multiple metrics of interest, a common approach is to determine weights based on expert judgment or domain-specific user knowledge [38,39]. Since the weights are incorporated linearly into the objective function, regression analysis or machine learning techniques can be highly effective for estimating their values when historical or experimental data is available. Additionally, in some cases, the weights may arise naturally during the development of the mathematical model [40].

The user experience of air quality monitoring is influenced by both the density of sensors in the monitoring area and the accuracy of pollutant detection. While high concentrations of hazardous substances may be present in the monitoring area, low pollutant levels near the sensor can make detection challenging. The spread of pollution is complicated by numerous unpredictable stochastic factors. To address these challenges, efforts are focused on improving sensor hardware and developing AI-based pollutant detection algorithms. For instance, air quality monitoring with a single sensor can be framed as a binary classification problem in machine learning, where the sensor determines whether pollutant concentrations exceed a predetermined threshold. The effectiveness of such sensors relies on both the quality of the hardware and the sophistication of the AI methods employed.

Thus, we conclude that it is necessary to minimize the penalty function for an individual sensor, taking into account the competing factors outlined below.

- *Pollution detection delay.* The harm caused by a delayed response to pollution increases with the time taken for pollution detection. The delay in detecting contamination can be modeled as a random variable following an exponential distribution with a rate parameter λp, where λ characterizes the sensor’s environment and *p* denotes the probability of detecting air pollution at a specified false negative rate [41].
- *Detection cost*. The effectiveness of AI-based air pollution detection methods often correlates with their cost, as higher accuracy typically require substantial computational resources. Machine learning enables us to achieve the desired performance even with low-cost sensors [42]. However, improving detection precision involves higher operational costs for training and maintaining AI models.

By choosing the mathematical expectation as the pollution detection delay metric and assuming that the cost of detection is linearly proportional to *p*, the problem of the penalty function minimization is formulated as follows:

(7) $$L\left(p\right)=\frac{c_{1}}{\lambda p}+c_{2}p$$

Here, the coefficient $c_{1}$ quantifies the economic, social, or operational cost associated with a unit increase in the expected pollution detection. The coefficient $c_{2}$ represents the incremental cost incurred by increasing the performance of air pollution detection method. This cost may include operational expenses such as sensor power consumption, maintenance, or increased resource allocation to improve pollution detection accuracy. In a given situation, the same principles mentioned above for selecting weights $w_{1}$ and $w_{2}$ apply to determining $c_{1}$ and $c_{2}$. Also, the coefficients can be derived directly by assigning monetary values based on accounting records or financial audit documents.

It is important to highlight that although the indicators mentioned above can be quantified in monetary terms, this approach may not resonate with the priorities of the specialists managing air monitoring systems. In practice, cost is a decisive factor in only 70% of cases. Furthermore, it has been noted that the expense associated with a monitoring system does not always correlate positively with its effectiveness [43], and there is no universally accepted standard for measuring user satisfaction. Depending on the specific problem and research objective, distance can be expressed not only in conventional units of the metric system but also as a fraction of a reference threshold, in terms of resource utilization, or through other context-dependent metrics. The weights in the proposed objective functions are selected to render the terms dimensionless, thereby normalizing the heterogeneous nature of the monitoring system’s metrics. Thus, the objective functions and the parameters of the corresponding optimization problems are conveniently treated as dimensionless.

## 4. Results and Discussion

In this section, we provide solutions to the optimization problems (6) and (7), formulated as nonlinear programming problems. It is worth noting that the profit function (6) can be expressed in the following form:

(8) $$F_{P}\left(R\right)=G_{E}\left(R\right)-\gamma C_{S}\left(R\right)$$

Here, we use the following designation:

(9) $$\gamma =\frac{w_{2}S}{w_{1}}$$

Maximizing (6) is equivalent to maximizing (8) because γ encapsulates the relative importance of user satisfaction to structural system cost, and scaling the objective by $w_{1}$ does not affect the solution. Maximizing (6) is equivalent to maximizing (8) because γ captures the relative importance of user satisfaction in relation to the structural cost of the system. Scaling the objective function by $w_{1}$ does not influence the optimal solution, as it simply applies a positive proportional factor. Objective functions of the form (8) are frequently encountered in the literature, where the balancing factor is typically assumed to be predefined [44,45,46].

Thus, we consider the following optimization problem:

(10) $$\max_{R}F_{P}\left(R\right)$$

We obtain:

(11) $$\frac{dF_{P}\left(R\right)}{dR}=\frac{Re^{-R}+e^{-R}-1+\gamma }{R^{2}}$$

Applying the necessary optimality condition:

(12) $$\frac{{dF}_{P}\left(R\right)}{dR}=0$$

we obtain the following equation:

(13) $$e^{-R}\left(R+1\right)=1-\gamma$$

This equation can be rewritten as follows:

(14) $${-(R+1)e}^{-(R+1)}=\frac{\gamma -1}{e}$$

Applying the Lambert *W*-function to both parts we obtain:

(15) $$R=-W\left(\frac{\gamma -1}{e}\right)-1$$

Since the distance R≥0, it follows that the value of Lambert *W*-function is less than −1. It is also necessary to take into account the natural limitation γ∈(0;1). Therefore, we focus on the lower branch of the Lambert W function, denoted as $\;W_{-1}$. Thus, the optimal value of cluster radius $R^{*}$ and the optimal number of clusters are as follows

(16) $$R^{*}=-W_{-1}\left(\frac{\gamma -1}{e}\right)-1$$

(17) $$n^{*}=\left\lceil \frac{S}{R^{*}}\right\rceil$$

Table 2 shows the optimal values of the clusterradius for different values of the parameter γ. The following trend of the optimal cluster radius behavior can be seen. As the parameter γ decreases, the radius smoothly decreases, while even a slight increase in the parameter γ, if its value is in the vicinity of 1, leads to a sharp increase in the optimal cluster radius.

**Figure 2:**
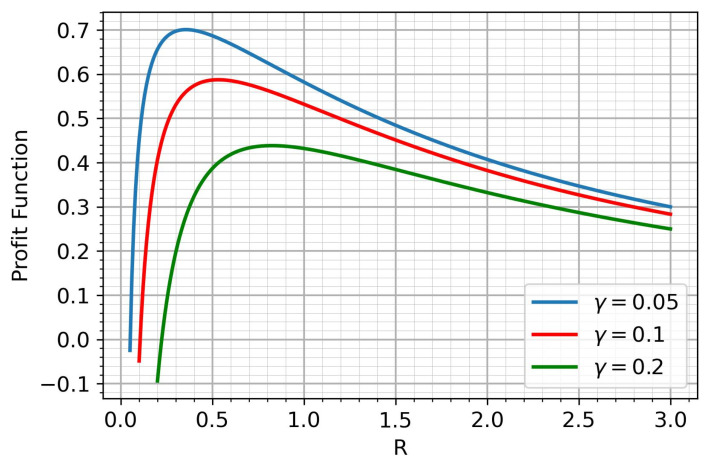
The profit function.

Under reasonable assumptions about the parameters of the income function, it has a unique maximum. Figure 2 shows the behavior of the income function for different S.

**Figure 3:**
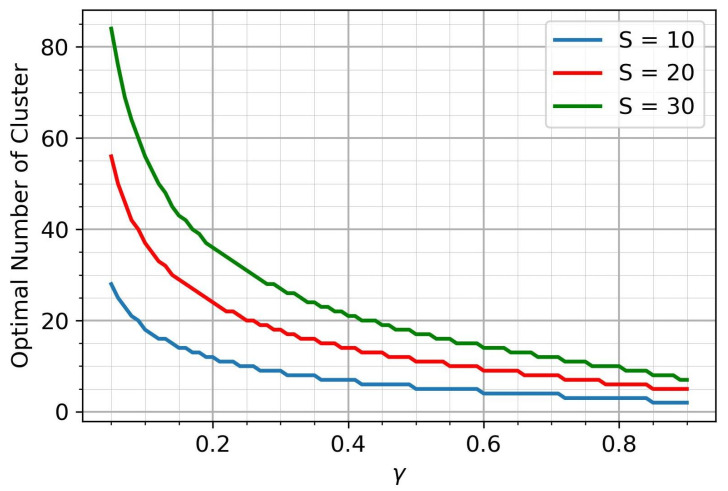
The optimal number of clusters.

The optimal number of clusters is a natural number, which means that its dependence on the parameter γ is a step function. Figure 3 shows the corresponding plots for various *S*.

We proceed similarly in the case of optimization of the penalty function

(18) $$\min_{p}L\left(p\right)$$

and obtain the optimal requirement for the performance of the AI-based air pollution detection method:

(19) $$p^{*}=\sqrt{\frac{c_{1}}{c_{2}\lambda }}$$

For the second derivative of the penalty function we have:

(20) $$\frac{dL(p)}{dp}>0$$

**Figure 4:**
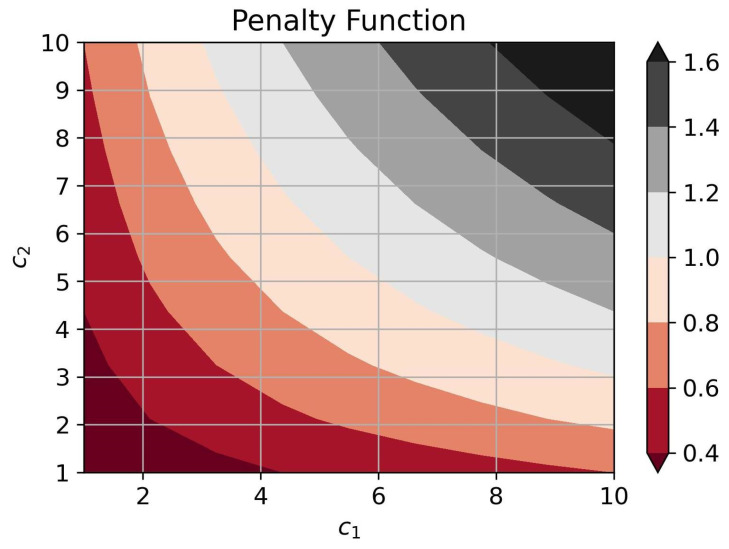
The penalty function behavior.

This guarantees a unique minimum of the penalty function. Figure 4 shows the behavior of the penalty function at the optimal performance of detection algorithms for different combinations of cost factors. Here, λ=100.

## 5. Conclusions

In recent years, substantial progress has been made in fundamental research, not only in understanding the propagation and transformation of natural and anthropogenic impurities in the atmosphere but also in the development of advanced air quality monitoring systems. Modeling has become an indispensable tool for both research and the formulation of air quality control strategies aimed at mitigating negative environmental impacts. Addressing the complex and interdisciplinary challenge of maintaining adequate air quality requires the establishment of effective environmental monitoring systems, including the optimization of their design and operation. This article presents a novel approach to optimizing the costs of air pollution monitoring systems, tailored to meet the needs of their users. It derives an analytical solution for determining the optimal number of sensors, expressed using the Lambert W function. To enhance the efficiency of real-time air pollution detection, the integration of AI-based solutions is recommended. The article further proposes a method for evaluating the performance metrics of these AI-driven systems, offering a pathway toward more responsive and effective air quality management.

Our future work will focus on developing and analyzing models not only for the deployment and management strategies of air monitoring systems but also for addressing operational challenges. The methodology will draw on multi-criteria optimization, traffic theory, and random graph theory to propose comprehensive approaches for optimizing air monitoring systems. These approaches will account for several additional factors, including the quality of service within the data collection network, segmentation of system performance metrics based on the application context, node unreliability, and the potential impact of uncertainty in measuring monitored parameters on decision-making processes.

## Figures and Tables

**Table 1:** Related works. Optimizing monitoring systems.

References	Description	Technique
Sun, C., et al. (2019) [6], Ajnoti, N. (2024) [7]	Optimal sensor placement: Citizen-Centric air quality monitoring. Maximizing overall satisfaction from receiving public information about air quality while limiting budget. Assumption of equal distance to the sensor for all cluster users. The statement of the optimization problem appears to be flawed.	Approximate optimization method (greedy algorithm, genetic algorithm).
Samudra, A.A., et al. (2024) [15]	Identification of the main sources of air pollution, quantitative assessment of their impact.	Simulation.
Mofarrah, A., et al. (2010) [16]	The statement of the optimization problem appears to be flawed. The optimal solution has not been rigorously defined.	Triangular fuzzy numbers.
Henriquez, A., et al. (2015) [17]	Three quality indicators are taken into account: accuracy gain, information gain, and degrees of freedom for the signal. The cost factor is not taken into account.	Heuristic optimisation algorithm – simulated annealing.
Huang, Z., et al. (2019) [18]	Optimizing an observation performance score that combines averages from both pollution detection effectiveness and source identification accuracy.	Merging Gaussian matching models with source area analysis.
Gupta, S., et al. (2018) [19]	Placement of monitoring stations.	Spatial simulated annealing method.
Gagliardi, G., et al. (2024) [20]	Problem of selecting a subset of sensors from a pool of potential sensors for monitoring a transportation network.	Simulated annealing heuristic, genetic algorithm.
Borges, M., et al. (2023) [21]	The optimization problem is not formalized. The objective function does not include the system cost metric.	Genetic Algorithm.
Mano, Z., et al. (2022) [22]	Optimal placement of sensors in known locations. The cost factor is not taken into account.	The Lagrange atmospheric dispersion model under different meteorological conditions. Then, sensors are deployed at the locations that are determined to be the most informative.
Liu, B., et al. (2023) [24]	Air quality monitoring network design problem with consideration of uncertain operational efficiency and wind conditions.	Maximum weighted location coverage problem is solved. Then, the linear approximation method is applied to the nonlinear model to improve the computational efficiency.

**Table 2:** Optimal cluster radius.

γ	$R^{*}$
0.01	0.149
0.1	0.532
0.99	6.638
0.999	9.233

## Data Availability

The data used to support the results of this study are included in the article.
